# Notes and News

**Published:** 1887-09-17

**Authors:** 


### Notes and News.

Collected and Communicated.

Mr. M. Biddulph, of London, gives £50 towards the new children's ward at the Hereford Infirmary.

On Sunday the first public collection was taken at Ilfracombe and in the neighbourhood, on behalf of the Tyrrel Cottage Hospital.

We give an article this week by Mr. Burdett on the National Pension Fund, the substance of which appeared in *The Times* on the 9th inst., over his signature.

Sir Andrew Clark, Bart., M.D., has consented to take the chair at the first evening meeting of The Hospitals Association for the ensuing session, when a paper will be read and details given of the scheme for the National Pension Fund for Hospital Officials and Nurses. The meeting will take place on Wednesday, October 12th, at 8 p.m., and we hope all who are interested will keep that evening free, to enable them to attend and take part in the proceedings. Further particulars will be published next week.

Mr. Cursham Corner, Visiting Medical Officer to the Tower Hamlets Dispensary, advises the treatment of scarlet fever cases at home, when possible, in preference to removing them to the hospital. He argues (1) that when a case is removed, people assume that infection has ceased, and go about their usual business, spreading contagion far and wide: they also use very inadequate methods of disenfection in such cases; (2) the knowledge, gained in treating fever at home, under proper advice induces increased care in preventing the spread of the disease, even in the homes of the poor.

At the International Medical Congress at Washington, after the delivery, without notes, of an address by Dr. Semmola on Scientific Medicine and Bacteriology, the whole audience rose *en masse* in token of its enthusiastic appreciation of the lecturer.

"Nell," a Tunbridge Wells dog, acting under the auspices of the United Friendly Societies, took part in their annual parade and collection on Sunday, with such goodwill that she herself collected no less than £2 8s. 6d. The whole of the proceeds, less expenses, were given to the Tunbridge Wells Hospital.

Guessing competitions are the last new method we have seen tried for raising the wind on behalf of hospitals. A grand *fête* has been arranged for at Rochdale, and one of the features of the entertainment will be a guessing competition, and it is said that between five and six thousand tickets for this event have already been sold. The sale of tickets is still going on with great rapidity.

Sir Spencer Maryon-Wilson, Bart., opened his park on Saturday for the holding of a grand *fête* and demonstration in aid of the funds of the proposed Cottage Hospital at Woolwich. Amusements of every kind were provided, including the Kentucky Minstrel Troupe, and a variety entertainment under the management of Mr. G. J. Stacey. Notwithstanding that a charge for admission was made, upwards of seven thousand people took part in the demonstration.

The town of Horncastle, as we noted some time ago, seemed inclined to hang back from its duty towards the Lincoln County Hospital. It might have been thought that those who were so slow to help the hospital had some local charity which was putting strain on their resources. We find, however, that the collections at the various places of worship made on behalf of the local dispensary this year have fallen off, and that there is a diminution of £3 in the total amount collected. Will any inhabitant of Horncastle inform us whether there be an unusual want of prosperity in the town, or whether this unpleasant result arises from coldness of heart and indifference to the interests of the sick poor?

The first year of the Brighton Dental Hospital's existence has just come to a close. The two or three main points of income, expenditure, usefulness, and so forth, are, according to the secretary's report, all satisfactory. Many places treat a new hospital like a new toy or a new baby. For the first brief period of its life it is in high favour, and receives all the help and nursing which a fond parent or possessor can bestow. Then the fickle town, like a faithless lover, begins to display symptoms of indifference and cold neglect. There is a way of keeping lovers and hospital supporters in a state of constant and admirable fervour. The loved object must always be pleasant, always intelligently active, always useful, and always improving. We hope the Brighton people will be true to their love, and we trust that the Dental Hospital will be so conducted that they cannot help it.

THE following vacancies are announced this week, the amount of the salary being placed in each case after the name of the institution :—

**Resident Medical Officers.**

Bristol Royal Infirmary. Assistant Resident Officer.—£80.
Devon and Exeter Hospital. House Surgeon—£120.
Female Lock Hospital, Harrow Road. House Surgeon.—£100.
St. Luke's Hospital. Clinical Assistant.

**Non-resident Medical Officers.**

Great Northern Central Hospital. Obstetric Physician.
Sheffield Infirmary. Two Honorary Assistant Physicians.
University of Aberdeen. Examiners in Science and Medicine. Six.

**Matron.**

Newport Infirmary and Dispensary.—£50.

**Trained Nurses.**

Central London Throat and Ear Hospital.—£20.
Fever Hospital, Darlington.—£25 and uniform.
South-Eastern Hospital, New Cross.—£30 to £36. Assistant, £22 to £26.
South-Western Hospital, Stockwell. Day and Night Nurses, £30 to £36. Assistant, £22 to £26.
Western Hospital, Seagrave Road, Fulham.—£30 to £36. Assistant, £22 to £26.

---

THE Hon. and Rev. Canon Freemantle gave an address at the annual visitation of the Royal Sea-bathing Infirmary, Margate, last Monday. On an average, about 750 patients are yearly relieved at this hospital, besides out-patients ; but it is a matter of much regret that, notwithstanding the appeals which are constantly being made for increased support, the income still falls considerably short of the expenditure, and the deficiency has to be made up by drawing upon the small amount of invested capital.

---

TILLICOULTRY does not despise the day of small things. A hospital appears to be needed for the district, and an attempt is being made to establish one. The cost will probably not amount to more than £500. Money does not seem to be lacking, nor public spirit, but, strange to say, in a district where houses are few and land is abundant, there is a difficulty about the site. Mr. Wardlaw Ramsey, the principal proprietor of the neighbourhood, is expected to visit the town shortly, when it is hoped the problem of a site will be solved.

---

VOLUNTEER collectors on behalf of Hospital Saturday at Belfast are less plentiful this year than on former occasions. At a meeting to arrange for the collection, at which the Rev. J. C. Street presided, this difficulty was considered, and the chairman hoped that in the event of the numbers not being adequately recruited, those who undertook the collection would by their increased energy make up for the want of service on the part of the defaulters.

---

THERE is a dispute at Folkestone with regard to the site of the proposed Victoria Hospital. Mrs. Ada Sturt offers £1,000 if the committee will give up a site on which they appear to have partly fixed their minds, and choose another. The committee, on the other hand, do not see their way to gratifying Mrs. Sturt, and the £1,000, we presume, is therefore still trembling in the balance. We cannot but think that the committee will be guided by wise counsels, and do that which is best for the hospital.

MR. CARR-GOMM presided on Wednesday afternoon at a Quarterly General Meeting of the Governors of the London Hospital. There were present Messrs. Morris Abrahams, F. Abrahams, J. Hall, Green, Plumpton, C. Lacey, Howlett, Saville, Hodsell, Shepherd, Carr, Bligh, Croger, and the Rev. Father Gorman. Several legacies and gifts were announced, amounting in all, including a grant of £3,333 6s. 8d. from the Hospital Sunday Fund, to £9,968 6s. 8d.

---

THE annual dinner of the old students of St. Thomas's Hospital will take place on Saturday, October 1st., in the Governors' Hall. The list of stewards closes on September 20th.

---

LAST week we reported a case of successful collecting of money by a dog at the West End. Mr. J. Bates, of Croydon, has also a clever dog which recently collected 15s. 4d. for the funds of the Croydon General Hospital.

---

SALISBURY is late in the day with its Hospital Sunday collection, but perhaps the fact that it is to be associated with the Harvest Festival makes this period of the year more suitable than any other for that city.

---

THE Royal Cornwall Infirmary, like its sister institution at Colchester, is said to be without a physician, and it appears that the work of the hospital is carried on to a large extent by the house-surgeon. This is surely a case demanding prompt and searching inquiry.

THE Pudsey Football Club gave an open-air concert in aid of the funds of the Leeds Infirmary and Convalescent Home. There was a full band, and a chorus of 400 performers, under the conductorship of Mr. H. Robertshaw. Nearly 10,000 persons took part in the proceedings.

---

THE Workmen's Industrial Exhibition at Sunderland has just closed its accounts. The balance-sheet shows that the total receipts amounted to £1,014 8s. 8½d., and the expenditure to £614 8s. 8½d., leaving a net sum of £400. This amount, by the vote of the managers of the Exhibition, has been handed over to the treasurer of the Sunderland Infirmary.

---

WE are officially informed that our note in last week's HOSPITAL, on the collapsed condition of the Glasgow Medical Charities Committee, was premature, and that that body, in common with all the rest of Glasgow, has been merely taking its annual holiday "doun the watter" or elsewhere. It has no intention of abating its exertions, and is quite unanimous. This is welcome news. Our rather sharp note was prompted by a feeling of disappointment at what appeared to be the entirely barren results of the committee's labours. There is, it is said, great apathy both among medical men and the public as to the abuse of medical charities. It is all the more necessary, therefore, that the committee should bestir itself until, by rousing arguments and indisputable facts, it dispels the apathy and establishes complete sympathy in its place. We shall hope to hear of good work being done before the end of the year.

It is proposed to build at Chichester a new hospital for infectious diseases.

---

The annual meeting of the managers of the Dunoon Cottage Hospital has just been held. The income exceeded the expenditure by nearly forty pounds.

---

The *Daily Chronicle* of Saturday had a very foolish and ignorant article on sick-nurses. Among other things, it spoke of "the so-called trained nurse" who is supplied by private enterprise, and who is, as a rule, simply a servant girl with a little hospital training combined with a great capacity for giving herself airs, and ordering special delicacies for her own delectation." This looks like sheer spite; or is it written by or in the interests of somebody who has "an axe to grind"?

---

A correspondent writes, with reference to our remarks on the falling off in the subscriptions to the Barton-under-Needwood Cottage Hospital, that the diminished income of the hospital is due principally to deaths and removals, and in no way to any want of interest or sympathy displayed by the ladies of the town and neighbourhood. It may be hoped, however, that the ladies and other subscribers will see their way to making some additional effort on behalf of the hospital, in consideration of the present peculiar circumstances.

---

The Rev. Alexander Robertson, of San Remo, writing to the *United Presbyterian Magazine*, describes the condition of the Catholic and Protestant patients in the Civil Hospital at Venice. Speaking of the wards in which the Catholic patients were lying, he says:—"Such infirmary wards we had never before seen. Their length, breadth, height, and embellishments are wonderful. The first we were taken into contained fifty beds; it might have held double or triple that number. . . . Altogether there are fifty such, with accommodation for 1,500 patients. All are airy, spacious, and well lighted; and the floors, furniture, and pretty, soft white-covered beds are of the most exquisite cleanliness. The patients are well cared for, and look contented, as well they might be." After seeing these beautiful wards, Mr. Robertson inquired if there were any Protestant patients, and finding that there were, asked to be allowed to see them. The Protestants have a separate ward, which he thus describes:—"What a change! Instead of fifty beds where a hundred could be, here are twenty in a space fit but for ten. The very beds themselves were inferior. No more soft spring-mattresses and white coverlets, but bedding of rough maize leaves and coloured cotton workhouse-looking quilts." Since our mission is to do what in us lies to unite all men in a common brotherhood of charity, we refrain from comment further than to express our deep regret that any public body should so misunderstand its duty as to make possible such a condition of things as that described by Mr. Robertson. Without any dread of being called pharisaic, we can safely affirm that nothing of the kind could happen in this country.

The scarlet fever outbreak is assuming alarming dimensions. The number of cases under treatment at the hospitals of the Metropolitan Asylums Board is approaching 1,100, whilst the whole of the available beds in all those hospitals do not reach 1,300. The managers of the Board met on Saturday, and the chairman, Sir Edwin Galsworthy, moved that it was high time to open the convalescent institution at Winchmore Hill, and thereby to render available a large number of beds occupied by those who have passed the acute stages of the fever. The motion was negatived by a small majority, and the Board will not meet again for a fortnight from the date of its last meeting. It is quite evident that many of the members of the Board have no understanding of the possibilities of the situation. If there should be, as is not at all improbable, any sudden and rapid extension of the disease, the Board will be powerless to receive the cases; and the consequence will be, that they may be left in hundreds to act as centres of infection, and to increase the spread of the fever tenfold. So long as the Asylums Board can effectually isolate most of the cases occurring in crowded districts, so long may it be hoped that the spread of the disease will be kept within manageable limits. But with all their resources cut off by the extraordinary decision of Saturday, it need occasion no surprise if the mild and limited outbreak of fever should assume the proportions of a formidable and uncontrollable epidemic.

---

The hospital at Bury St. Edmunds was reopened on Thursday, after having been closed for repairs, and for covering the walls of two wards with glazed tiles. Lady Alwyne Compton declared the wards open, and a large and fashionable company assembled at the ceremony. The cost of the improvements, amounting to £450, has been defrayed by a Jubilee subscription.

---

Times are evidently bad at the Devon and Exeter Hospital. By a vote of sixteen against nine, the governors have resolved to diminish the salary of the house-surgeon by £30 a year, from £150 to £120. Nine wise men desired to keep the salary up to £150, justly reasoning that they would for that sum probably obtain a much better man; but the sixteen were immovable. They may, however, be assured that, in the open market, house-surgeons are exactly the same as red herrings—the quality is in strict proportion to the price.

---

The *North Middlesex Chronicle* says:—"The garden party at Stamford Hill, in aid of the Metropolitan Hospital, Kingsland Road, calls to mind a melancholy fact that is becoming more apparent ever day. . . . . Originally the above institution was entitled the 'Metropolitan Free,' but the latter word is now dropped, and its chief feature is therefore gone. Some time back Sir Edmund A. Currie was elected hon. sec., but not being able to agree with his committee he resigned, and the management of the hospital seems to have gone from bad to worse, until the original idea appears to be discarded, and the hospital to be free no longer."

